# Fatal accidental lipid overdose with intravenous composite lipid emulsion in a premature newborn: a case report

**DOI:** 10.1186/s12887-021-03064-6

**Published:** 2021-12-20

**Authors:** Maliha Badr, Marion Goulard, Bénédicte Theret, Agathe Roubertie, Stéphanie Badiou, Roselyne Pifre, Virginie Bres, Gilles Cambonie

**Affiliations:** 1 Department of Neonatal Medicine and Paediatric Intensive Care, Arnaud de Villeneuve Hospital, Montpellier University Hospital, University of Montpellier, 371 Avenue du Doyen Gaston Giraud, 34295 Montpellier Cedex 5, France; 2 Department of Neuropaediatrics, Gui de Chauliac Hospital, Montpellier University Hospital, University of Montpellier, Montpellier, France; 3 Department of Biochemistry and Hormonology, Lapeyronie Hospital, Montpellier University Hospital, University of Montpellier, Montpellier, France; 4 Department of Medical Pharmacology and Toxicology, Montpellier University Hospital, University of Montpellier, Montpellier, France; 5 Pathogenesis and Control of Chronic Infection, INSERM UMR 1058, University of Montpellier, Montpellier, France

**Keywords:** Medical errors, Newborn, Lipid overdose, Exchange transfusion, Lipid emulsions

## Abstract

**Background:**

Tenfold or more overdose of a drug or preparation is a dreadful adverse event in neonatology, often due to an error in programming the infusion pump flow rate. Lipid overdose is exceptional in this context and has never been reported during the administration of a composite intravenous lipid emulsion (ILE).

**Case presentation:**

Twenty-four hours after birth, a 30 weeks’ gestation infant with a birthweight of 930 g inadvertently received 28 ml of a composite ILE over 4 h. The ILE contained 50% medium-chain triglycerides and 50% soybean oil, corresponding to 6 g/kg of lipids (25 mg/kg/min). The patient developed acute respiratory distress with echocardiographic markers of pulmonary hypertension and was treated with inhaled nitric oxide and high-frequency oscillatory ventilation. Serum triglyceride level peaked at 51.4 g/L, 17 h after the lipid overload. Triple-volume exchange transfusion was performed twice, decreasing the triglyceride concentration to < 10 g/L. The infant’s condition remained critical, with persistent bleeding and shock despite supportive treatment and peritoneal dialysis. Death occurred 69 h after the overdose in a context of refractory lactic acidosis.

**Conclusions:**

Massive ILE overdose is life-threatening in the early neonatal period, particularly in premature and hypotrophic infants. This case highlights the vigilance required when ILEs are administered separately from other parenteral intakes. Exchange transfusion should be considered at the first signs of clinical or biological worsening to avoid progression to multiple organ failure.

**Supplementary Information:**

The online version contains supplementary material available at 10.1186/s12887-021-03064-6.

## Background

Medication error is one of the leading causes of safety incidents in neonatal intensive care units (NICUs) [1]. An estimated 1–8% of these iatrogenic events is life-threatening [2], most often resulting from errors in prescribing or programming the flow rates of electric infusion pumps by tenfold or more [3–6]. Among neonates hospitalized in NICUs, the patients most at risk are those with the lowest gestational age and birthweight, the greatest severity of illness on admission, and a prolonged length of stay [7–9]. The most dangerous drugs include insulin, cardiovascular drugs, sedatives, and electrolytes [6, 10, 11]. Intravenous lipid emulsion (ILE) infusion is also a high-risk medication, notably if lipid emulsion is infused separately from the other components of parenteral nutrition (PN). Accidental lipid overdose due to an excessive infusion rate of ILE may result in serious complications, such as respiratory failure, metabolic acidosis and even death. Treatment of lipid overdose in newborns is not standardized. Recovery after exchange transfusion (ET) has been described in rare case reports, but it remains a high-risk procedure in sick preterm infants [12, 13]. We report an exceptionally serious adverse event following the administration of a composite intravenous lipid emulsion (ILE) and discuss the mechanism of the medication error and the pathogenesis and management of lipid overdoses in neonates.

## Case presentation

This boy, born at 30^+ 1^ weeks of gestation to a 32-year-old mother, was the first child of non-consanguineous Caucasian parents. The mother was overweight before pregnancy, with a BMI of 30. Elevated maternal serum biochemical markers in the first trimester of pregnancy prompted a prenatal noninvasive test for trisomy 21, which was negative.

The mother consulted at her local hospital, a type 2 perinatal centre, for headaches that had worsened over 48 h. The examination found arterial hypertension (145/95 mmHg) with no other sign of preeclampsia. Foetal heart rate monitoring was non-reassuring, with reduced variability and decelerations. Ultrasound and Doppler assessment showed decreased active foetal movements, absent diastolic flow in the umbilical artery, and cerebral vasodilation (resistance index = 0.5). Intramuscular betamethasone (12 mg) was administered to the mother, and caesarean delivery was decided on 1 h later in a context of more pronounced decelerations.

Apgar scores were 4/7/10 at 1, 5 and 10 min, respectively; arterial cord blood pH was 6.97 and cord lactate was 16 mmol/L. Birthweight was 930 g (<3rd centile, according to Olsen curves [14]), and head circumference was 25.5 cm (3rd-10th centile). Pathological examination of the placenta found four foci of infarction, with size varying between 7 and 12 mm in the major axis, representing < 10% of placental volume. Examination outside these areas was considered normal. The neonate was bagged with 30% oxygen for a few minutes and then supported with nasal continuous positive airway pressure (CPAP). Peripheral venous catheterization was performed to provide standard hydration, vitamin K (1 mg), and a bolus dose of caffeine (20 mg/kg). The newborn was then transferred to a type 3 NICU.

On admission, 3 h after birth, the fraction of inspired oxygen (FiO2) required to maintain adequate oxygenation had increased to 70%, prompting surfactant administration (200 mg/kg of poractant alfa), according to the less invasive surfactant administration procedure with propofol (1 mg/kg) for premedication, as stated in our service protocol [15]. Soon after, the FiO_2_ required to maintain adequate oxygenation decreased to 25% and capillary blood gases showed improved pH (7.28) and lactate (6.8 mmol/L) compared to cord blood values. An epicutaneocaval catheter was inserted into the left basilica vein at the 7th postnatal hour to perfuse PN with a separate glucose-amino acid solution (2.7 ml/h of Pediaven NN2, Fresenius Kabi, Sèvres, France), an additional solute of amino acids (0.3 ml/h of Primène 10%, Baxter, Maurepas, France), and ILE (0.2 ml/h of Medialipide 20%, B. Braun Medical, Boulogne, France), the whole providing 7 g/kg/day of carbohydrates, 2 g/kg/day of proteins, and 1 g/kg/day of lipids, with carnitine (8 mg/kg), multivitamins (Cernevit, Baxter, Maurepas, France) and trace elements (Nutryelt, Aguettant, Lyon, France). Enteral feeding was initiated, with 16 ml per day of donor human milk administered continuously through a gastric tube. Thrombocytopenia (33,000/mm^3^, see reference ranges for newborns and infants used by our laboratory in the Table (Table 1)) and impaired coagulation tests (prolonged prothrombin time – with a decrease in all vitamin K-dependent factors – and partial thromboplastin time, fibrinogen < 0.35 g/L) prompted the administration of 20 ml/kg of fresh frozen plasma.

**Table 1:** Reference ranges for newborns and infants used by our laboratory

Glucose, mmol/L	Days 0–1: 2.2–2.7 Usual values in premature newborns with parenteral nutrition: 2.6–9.9	Days 2–30: 2.8–4.4
Creatinine, μmol/L	Days 0–1: 52–86 Day 3: 40–69 Day 7 and above: < 53	Day 2: 47–82 Day 4: 36–66
Sodium, mmol/L	Days 0–30: 133–146	
Potassium, mmol/L	Days 0–30: 3.5–6.0	
Bicarbonate, mmol/L	22–29	
ASAT, UI/L	First year: < 58	
ALAT, UI/L	First year: < 41	
Creatine kinase, UI/L	Days 0–1: < 712	Days 2–5: < 672
LDH, UI/L	Days 0–14: < 1128	
Protein, g/L	Days 0–14: 51–80	
Albumin, g/L	Days 0–5: 28–44	
Calcium, mmol/L	Days 0–10: 1.80–2.80	
Phosphate, mmol/L	Days 0–14: 1.1–2.8	
Triglycerides, g/L	First year: 0.52–2.31	
Hematocrit, (%)	Days 0–30: 35–65	
Neutrophil, /mm3	Day 1: 7200-12,600 Day 3: 1800–7000	Day 2: 4200-9000 >Day 5: 1800-5400
Platelets /mm3 Giga/L	150,000-400,000 150–400	
Fibrinogen, g/L	Days 0–6: 1.5–3.7	
C-reactive protein, mg/L	Days 0–30: < 5	
pH	Days 0–1: 7.29–7.45	Days 2–30: 7.35–7.45
PaCO_2_, mmHg	Days 0–30: 35–45	
PaO_2_, mmHg	Days 0–30: 55–65	
Lactate, mmol/L	Days 0–1: 1.5–5.0	Days 2–30 < 2.9
Free carnitine, μmol/L	Premature: 11–63	Full term: 20–52
Common coagulation test		
PT, s	Premature: 15–30	Full term: 12–23
PTT, s	Premature: 30–80	Full term: 30–55
Fibrinogen, g/L	Days 0–6: 1.5–3.7	
II, %	Premature: 15–50	Full term: 27–64
V, %	Premature: 23–70	Full term: 50–140
VII, %	Premature: 31–62	Full term: 21–65
X, %	Premature: 16–36	Full term: 28–78

*ALAT* alanine aminotransferase, *ASAT* aspartate aminotransferase, *LDH* Lactate dehydrogenase, *PT* prothrombin time, *PTT* partial thromboplastin time

Twenty-four hours after birth, the respiratory state was stabilized with CPAP + 6 cm H_2_O and FiO_2_ 24%; haemodynamics was adequate with heart rate: 152 bpm, mean arterial blood pressure: 35 mmHg, and diuresis: 5.2 ml/kg/h over the first 24 h. Five episodes of hypoglycaemia, ranging between 1.4 mmol/L and 2.5 mmol/L and occurring after PN initiation, had required 10% glucose intravenous boluses. The PN prescription was changed to (i) a preparation in the department of a mixture of glucose, proteins and electrolytes corresponding to respective glucose and protein intakes of 10 g/kg/day and 3 g/kg/day, infused at a rate of 4.1 ml/h, and (ii) continuation of the same ILE at a rate 7 ml/day, i.e. 0.3 ml/h, corresponding to lipid intake of 1.5 g/kg/day. The exact wording for the parenteral nutrition over the next 24 h was: (i) Preparation (with details on the different solutes of the mixture): total 98 ml, at a flow rate of 4.1 ml/h, and (ii) Medialipide 20%: 7 ml, given separately from the main mixture. The prescription, however, was misinterpreted by the nurse, who administered ILE at a rate of 7 ml/h. The error was identified 4 h later by the physician who visited the infant for an increase in oxygen requirements. The infant was found to have tachypnoea, with a respiratory rate of 60–70 breaths per minute compared to 40–50 in the preceding hours. Other vital signs were normal, lungs were clear to auscultation, there was no cardiac murmur, and neurologic examination was normal. ILE was immediately stopped, but it was estimated that the infant had received 5.6 g of this emulsion, i.e. 25 mg/kg/min of lipids over 4 h, before cessation.

**Fig. 1:**
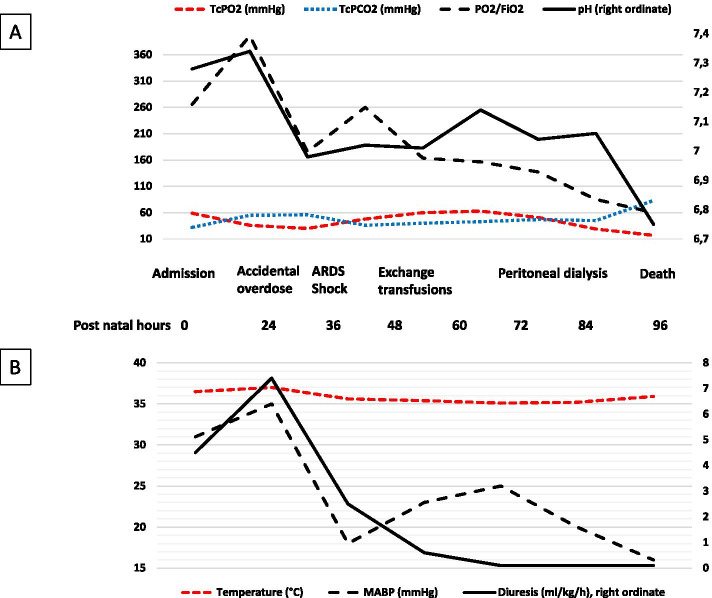
Clinical evolution. **A** Markers of acute respiratory distress syndrome (ARDS). TcPO_2_: transcutaneous oxygen pressure; TcPCO_2_: transcutaneous carbon dioxide pressure; FiO_2_: fraction of inspired oxygen. **B** Markers of acute shock. MABP: mean arterial blood pressure

**Fig. 2:**
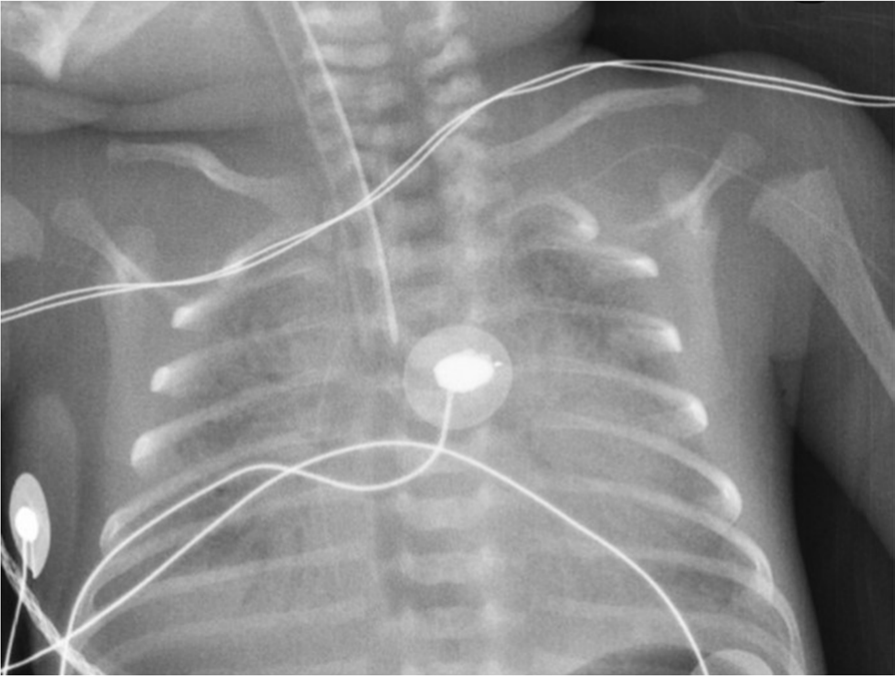
Chest X-ray, 10 h after the lipid overdose and patient intubation

The infant’s condition rapidly deteriorated. Four hours after stopping ILE, the infant was still tachypnoeic, and the FiO_2_ required to maintain adequate oxygenation had increased to 50%. The infant’s alertness, muscle tone and motor skills were preserved. Values for systolic, diastolic, and mean blood pressure (respectively 49, 27, and 35 mmHg) were comparable to those observed in the previous hours. Capillary blood gases showed pH: 6.98, PCO_2_: 56 mmHg, PO_2_: 45 mmHg, bicarbonate: 13 mmol/L, and lactate: 11.4 mmol/L. Echocardiography found markers of pulmonary hypertension (PH), with peak tricuspid regurgitant jet velocity reaching 3 m/s – for an estimated systolic pulmonary artery pressure of 35–40 mmHg − and a predominant right-to-left shunt in the ductus arteriosus. Nitric oxide (20 ppm) was administered on the inspiratory branch of the CPAP circuit, and transcutaneous blood gas monitoring was implemented. After a transient improvement lasting a few hours, with FiO_2_ reduced to 35%, acute respiratory distress syndrome developed (Fig. 1A). This required intubation and high-frequency oscillatory ventilation 10 h after the lipid overdose. Chest X-ray following intubation showed reticular infiltrates with a positive air bronchogram and reduced lung volume (Fig. 2), prompting the administration of a second dose of surfactant.

Shock occurred in the following hours, with collapsed blood pressure and anuria, refractory to continuous alkalization with 4.2% bicarbonate, albumin, norepinephrine, and an attempt to restart diuresis with furosemide (Fig. 1B). Successive echocardiographs while the infant was still being treated with inhaled nitric oxide showed persistent PH, as previously described, normal left ventricular function (fractional shortening 30–35%), adequate cardiac output (estimated at 200–250 ml/kg/min), and absence of cardiac thrombus or pericardial effusion. Bleeding in the gastric and tracheal tubes and at the entry of the epicutaneocaval catheter, associated with profound thrombocytopenia (< 10,000/mm3), prompted the transfusion of platelets, red blood cells, and fresh frozen plasma.

Seventeen hours after the end of lipid overload, biological assays revealed a very high serum triglyceride level, 51.4 g/L, moderate cytolysis (alanine aminotransferase: 91 IU/L, aspartate aminotransferase: 151 IU/L, creatine kinase: 1002 IU/L, lactate dehydrogenase: 1936 IU/L), renal failure (creatinine: 122 μmol/L), haemodilution (protein: 20 g/L, albumin: 11 g/L, sodium: 130 mmol/L), hyperglycaemia (26 mmol/L), hypophosphataemia (0.86 mmol/L), normal calcium (2.36 mmol/L), and increased lactate (12.2 mmol/L). Persistent thrombocytopaenia was associated with decreased neutrophil count (340/mm^3^). Coagulation tests could not be performed at this time point because of extreme lipaemia.

**Fig. 3:**
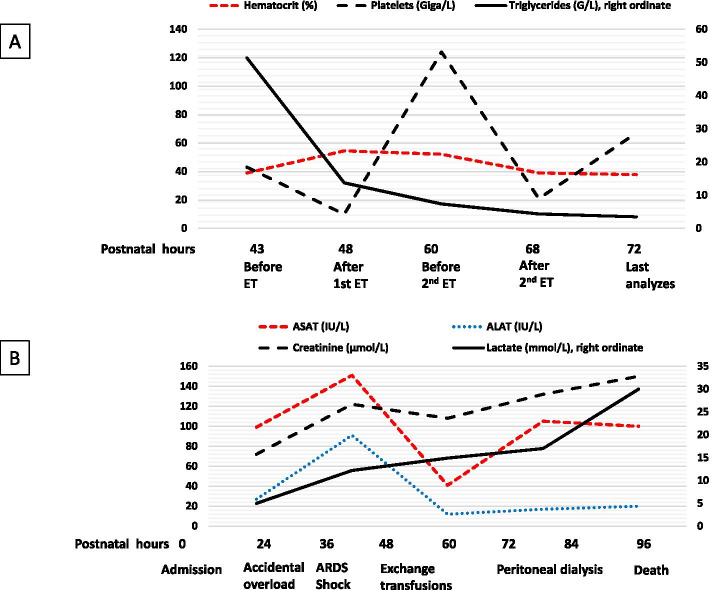
Biological evolution. Please note **A** and **B** have different time scales. **A** Effect of exchange transfusion (ET) on some biological parameters. **B** Biological markers during hospitalization. ALAT: alanine aminotransferase; ASAT: aspartate aminotransferase; ARDS: acute respiratory distress syndrome

The first exchange transfusion (ET) was started 5 h later, i.e. 22 h after stopping ILE, a delay required for the placement of a double lumen umbilical venous catheter and the reception of blood products. After the exchange of 210 ml, i.e. approximately a 3-volume exchange transfusion, this technique reduced the triglyceride level to 13.72 g/L. The same technique with the same exchanged volume was repeated a few hours later, and the last analysis 43 h after the lipid overload showed a reduction in the triglyceride peak by 14- to 15-fold (Fig. 3A).

The infant’s condition nevertheless remained critical, with persistent bleeding and shock despite repeated transfusions of platelets and fresh frozen plasma, catecholamines, the vasopressin analogue terlipressin and broad-spectrum antibiotics (cefotaxime, amoxicillin and amikacin). Glycaemia was constantly > 25 mmol/L despite continuous insulin infusion (0.1 IU/kg/h). A peritoneal catheter was placed, but the renal replacement therapy was ineffective in producing significant ultrafiltration and reversing oedema and acidosis. Two blood cultures and a peritoneal fluid culture did not identify any pathogens. Hypofibrinogenaemia (0.5 g/L), with prolonged coagulation times beyond the measured thresholds, was observed. Factors II, V, VII and X ranged from 11 to 19%. The infant died 96 h after birth, i.e. 69 h after lipid overload, in a context of refractory lactic acidosis (Fig. 3B).

A few blood tests could be carried out a posteriori on samples preceding the lipid overload kept in the biochemistry laboratory. The acylcarnitine profile found increased free carnitine (235 μmol/L) and increases in short- and medium-chain acylcarnitines, which could be attributed to supplementation. We observed no increase in long-chain acylcarnitines, potentially suggestive of carnitine palmitoyl transferase 1 deficiency, but classically observed with carnitine supplementation. Amino acid chromatography showed an increase in alanine and proline, as frequently observed in relation with lactic acidosis and birth asphyxia. In addition, a decreased ratio of branched-chain amino acids/(phenylalanine + tyrosine) was suggestive of hepatic dysfunction.

Parents were immediately informed of this serious adverse event associated with care. They did not want an autopsy to be carried out, but they gave their consent for whole-exome sequencing for themselves and their infant to search for abnormalities, particularly metabolic diseases, which might have favoured such a severe clinical expression of lipid overload. Whole-exome sequencing did not identify a pathogenic variant that could be related to the infant’s symptoms. The event was reported to the quality and risk management department of our institution, to the regional pharmacovigilance center and to the regional health agency. It was entered in the national pharmacovigilance database, for transmission to the European and global pharmacovigilance databases (EudraVigilance and VigiBase).

## Discussion and conclusions

We report a fatal accidental lipid overdose in a newborn infant, which for the first time involved a composite lipid emulsion. We could precisely document the cause of this iatrogenic event and the clinical and biological evolution, notably after ET, but the absence of an autopsy does not allow us to confirm the pathophysiological hypotheses about what had led to the patient’s death.

Adequate protein and energy intake through PN may improve postnatal growth, cognitive performance and neurologic outcome in premature infants [16–18]. According to the 2018 guidelines on paediatric PN, at least 45–55 kcal/kg/day should be provided on the first day of life of premature neonates [19]. It is also recommended that ILE start immediately after birth in this population, and no later than the second day of life [20]. The infant’s energy requirements were effectively met by PN; however, some peculiarities in the prescription are worth highlighting.

On postnatal day 1, the clinician prescribed a standard PN solution, in line with current recommendations [21]. Two types of commercially prepared standard PN for newborns are available in our institution: two-in-one (proteins and dextrose while lipids are given separately) or all-in-one (a bag containing protein, dextrose and lipids). The composition of these standard PN is integrated into the in-house computerized physician order entry system of our department [22]. The clinician chose the first option, supplemented by an amino acid solute, because the prescription summary proposed more satisfactory intakes in macronutrients, micronutrients and fluids. According to the recommendations for neonatal PN practice in our country, if required, a commercially prepared standard PN with one or more supplements is an option, but it is considered suboptimal [23].

On postnatal day 2, the clinician decided to increase carbohydrate intake, given the hypoglycaemia observed over the previous hours. As the newborn had been admitted on a weekend, an individualized PN formulation could not be prepared by the usual pharmaceutical subcontractor, as this establishment is closed during on-call periods. The clinician thus decided to prescribe an individualized PN using the department’s prescription software, cited above, that takes into account the total volume and the desired nutrient intakes. This indication of an individually tailored PN solution for a metabolically unstable patient is recognized in current recommendations, as is the use of computerized prescription for the PN ordering process [21].

Using a drug or preparation involves various caregivers and several steps, including prescription, preparation, and administration. A recent report, based on direct observation by a clinical pharmacist, showed that among the errors identified, 38% occurred during prescription, 16% occurred during preparation, and 45% occurred during administration [24]. In our department, when nurses receive a prescription for a patient, they perform three actions. First, they transcribe the prescription on their monitoring sheet, where all the drugs and solutions for parenteral nutrition are assigned an hourly flow rate. Second, they put labels on all syringes required for the prescription, with the name of the drug or preparation, as well as the hourly rates of administration. Third, they prepare the solutions or drugs to be administered to the patient and put them in the syringes they have previously labelled. The prescription was carried out according to our department standards, since the prescription of all solutions for parenteral nutrition applies for the next 24 h, but it lacked precision. To avoid any confusion, the prescriber should probably have specified not only the daily volume of the ILE, but also its hourly flow rate, as was specified for the mixture of glucose, proteins and electrolytes. This lack of precision in the prescription likely favoured an error in transcribing, since the prescription intended 7 ml/day of the ILE to be given separately from the main mixture, whereas the infant’s nurse monitoring sheet, as well as the flow rate noted on the label of the syringe containing the ILE indicated 7 ml/h. This error led to a second inevitable consequence, namely an incorrect flow rate, 24 times more than required over 4 h.

Many events occur almost uninterruptedly in a neonatal intensive care unit (NICU), which can alter nurses’ concentration during the preparation of solutions and drugs, even when the preparation area is separated from the central zone of the unit by a door, as is the case in our department. During the retrospective analysis of this adverse event, the nurse did not report having been disturbed during the three steps preceding the administration of the ILE. She pointed out, however, that she had truly wondered about the quantity and flow rates of the ILE and that she had questioned her colleagues when writing the flow rate on the label and during the preparation of the syringe. She also thought of obtaining medical confirmation of the flow rate when the syringe was placed on the infusion pump; however, the physician was at this moment attending to another patient. These factors suggest that the nurses of our department have insufficient knowledge and benchmarks on parenteral nutrition of the newborn [25]. However, educating the nursing staff is insufficient to reduce the risk of such medication errors. Other actions to be considered in our NICU notably include (i) better standardization in the prescription of parenteral nutrition with systematic specification of hourly flow rates, (ii) computer links between the medical prescription and the nurse monitoring sheet to avoid transcription steps as much as possible, (iii) the use of new-generation syringe pumps connected to the patient’s prescription file, which could include the programming of a drug library with pre-specified doses and hourly infusion rates, and (iv) adjustment of the medical workforce during on-call periods to cope with high levels of care activity and respond more promptly to nurses’ requests.

Our patient had received propofol, a molecule prepared in a lipid emulsion, for premedication before surfactant administration. His clinical course, and some pharmacological arguments, suggest that propofol administration probably had a negligible influence, if any, in the occurrence of clinical and laboratory symptoms. Pharmacodynamic data observed in neonates during laryngoscopy suggest respiratory and/or clinical recovery from sedation within 15–30 min in a large majority of infants after variable doses (1–2.5 mg/kg) of intravenous propofol [26–30]. From a biological point of view, the administration of 1 mg/kg of propofol corresponds to a very low lipid intake, 5 mg of soybean oil, which cannot explain the very high serum triglyceride level observed 39–40 h after propofol administration.

Adverse events associated with ILE are infrequent and usually non-life-threatening, with the exception of patients at the extremes of age, particularly neonates and infants [31]. Fat overload syndrome is characterized by an increase in triglyceride levels, associated with fever, cholestasis, hepatosplenomegaly, and coagulopathy. It has been reported in patients receiving ILE in high dosages [32, 33] and/or for extended periods of time [34, 35]. Another cause is accidental lipid overdose due to an excessive infusion rate. In the neonatal literature, four cases are reported with Intralipid 20%, a pure soybean oil emulsion [12, 36–38]. Medialipide is a 20% emulsion consisting of half medium-chain triglycerides and half soybean oil. Four other cases of overdose with this product following an administration error were identified both in the national pharmacovigilance database and VigiBase [39], including three premature newborns who presented mild and resolving symptoms after ILE had been stopped for some days. The most common symptom was hypertriglyceridaemia; transcient tachycardia and polypnoea were observed in one newborn. The last case involved a more extensive overdose in a 23-month-old male child with a severe pulmonary history. He rapidly presented a deterioration of the general condition with fever (39 °C), sweating, polypnoea, desaturation and acute respiratory distress, requiring transfer to a paediatric intensive care unit to provide respiratory support. The severe hypertriglyceridemia and the overall condition improved after 10 days [40].

One of the most consistently observed effects in ILE overdose is respiratory distress, which can be of multifactorial origin. The error in infusion rate can cause volume overload which, in our case, was one third of the expected daily volume with PN. Alteration in cerebral perfusion, potentially involving fat emboli in brain capillaries and arterioles [41], has been associated with lethargy and apnoea in premature infants [12, 13]. We did not observe these symptoms in our patient, who was instead described as uncomfortable and often crying as the demand for oxygen increased. The marked reduction in the PaO_2_/FiO_2_ ratio observed shortly after lipid overdose in our patient may be explained by hypertriglyceridaemia itself, as a decrease in oxygen-diffusing capacity through the alveolar-capillary membrane parallels the increase in plasma concentrations of triglycerides [42]. Indeed, autopsy series in premature infants have found fat accumulation in the pulmonary vasculature and capillaries engorged with large lipid globules, even after usual intakes of ILE [43, 44]. The aggregation of lipid microparticles and embolization of fat emulsion in the pulmonary circulation could be facilitated by high concentrations of C reactive protein [45]. In our patient, the C-reactive protein level was 1.6 mg/L 4 h before the accidental overload.

Another source of ventilation perfusion mismatch, clearly demonstrated in our patient, was the alterations in vascular tone, leading to PH, an increase in pulmonary vascular resistance, and ductal right-left shunting. Based on our experience, a peak tricuspid regurgitant jet velocity reaching 3 m/s was clearly high for this patient, corresponding to approximately double the velocity we usually record in premature infants [46]. Using a relevant M-mode echocardiography index to assess pulmonary haemodynamics, a study in stable low birthweight neonates suggested increased pulmonary vascular resistance during the infusion of 0.6 g/kg of a pure soybean oil emulsion over 2 h [47]. With the same instrument, dose- and time-dependent increases in pulmonary vascular resistance were found during continuous infusion of the same emulsion in premature infants with respiratory distress [48]. Of note, serum triglyceride concentrations were within normal ranges during this study, suggesting that changes in pulmonary resistance may be related to the production of vasoactive eicosanoid lipid metabolites derived from long-chain polyunsaturated fatty acids. Inhaled nitric oxide improved PaO_2_/FiO_2_ only transiently, suggesting that the sustained PH observed in our patient may indeed have involved a deregulation in the balance between circulating vasoconstrictors like thromboxane A2 and vasodilators like prostaglandin I2 [49].

Systemic haemodynamic deterioration may result from neonatal PH; however, it rarely manifests in immediate and severe shock, as observed in our case [50]. Repeated echocardiographs did not show signs of left ventricular dysfunction, left ventricular output was in the normal range [51], and bacteriological samples showed no evidence of septic shock, although neutropenia was transiently observed after lipid overload. Despite these haemodynamic features, tissue perfusion was clearly impaired, resulting in anuria and increasing lactic acidosis. Extensive fat sludging in the microvasculature of multiple organs is found in fat overload syndrome, and autopsy studies demonstrated a direct link between the accumulation of fat in a vessel and tissue necrosis in its perfusion territories [52]. End-organ dysfunction depends on the distribution of fat emboli, which in our case we speculate may have predominated in lungs and kidneys. In addition, the acidosis, recurrent bleeding, and coagulopathies suggested diffuse damage to capillary endothelium by sludged fat deposits. Insulin resistance may have been the consequence of an acute stress of the hepatic endoplasmic reticulum [53].

Treatment of lipid overdose in newborns is not standardized beyond stopping the intravenous lipid emulsion and providing supportive care. ET was introduced in the late 1940s to reduce the mortality of neonatal haemolytic disease and became one of the most common procedures in neonatology in the mid-90s. Experience with ET in premature newborns is limited, especially in the current period characterized by the sharp decline in the number of ETs performed over the last two decades [54]. This lack of familiarity thus also applies to our medical and nursing staff and potentially raises the hypothesis that the outcome of our patient might have resulted, at least in part, from an ET-related adverse effect. Several authors, however, have pointed out the uncertainty in ascribing complications to this technique in infants who were already very ill before it was started [54–58]. In our observation, ET was decided upon and then performed in a patient who already had ARDS and acute shock. In these conditions, the procedure was very effective in reducing triglyceride levels but provided no clinical benefit. This result is inconsistent with two case reports that reported dramatic improvement with this procedure. The first case was a 5-day-old premature infant born at 32 weeks’ gestation with a birthweight of 2160 g, whose triglyceride level was more than double what we recorded (129 g/L) [12]. The second was a twin born at 25 weeks and 6 days gestation, with a birthweight of 920 g, whose triglyceride level could not be measured [13]. In both cases, an ET with two blood volumes was performed, but the authors did not specify the delay between lipid overdose and ET completion. In addition, full recovery after single-volume blood exchange transfusion was reported in a case of accidental ILE overdose (serum triglyceride level 48 g/L), in a 3-month-old male infant [59]. The evolution of our case suggests that the clinical efficacy of this technique would have required more rapid implementation − perhaps at the observation of high blood lactate levels a few hours after the overdose − in order to avoid evolution towards multiple organ failure. Multiple vulnerability factors were present in our patient and were of antenatal and postnatal origin. These included intrauterine growth-restriction, incomplete antenatal corticosteroid course, perinatal asphyxia, caesarian section delivery, outborn delivery, extremely low birthweight, and very preterm infant birth. It can be speculated that the tissue vulnerability of this fragile neonate may have had a role in the unfavourable evolution. Indeed, the amino acid profile revealed that several markers of liver damage due to neonatal asphyxia, such as increased alanine and a decreased ratio of branched-chain amino acids/(phenylalanine + tyrosine), were already present before the ILE overload. In stable eutrophic premature newborns after a few days of life, a bolus of 1 g/kg over 15 min of a soy-based ILE increased, on average, triglyceride levels to 10 g/L, with the level then normalizing within 2 h of the beginning of infusion [60].

Rapid infusion of ILE has been proposed to treat the toxicity of local anaesthetics and various other drugs. The mechanism of action of ILE appears to be essentially related to the binding property of the emulsion. Different ILEs have been used, such as the one administered to our patient [61], and this strategy has also been successfully applied to the paediatric population [62]. Regional anaesthesia is used in about one third of all surgical procedures in neonates and has proven useful in improving the treatment of postoperative pain and reducing the use of systemic analgesics such as opioids [63]. While complications are rare and usually minor [64], this case report should prompt clinicians to take great care in using an ILE to resuscitate a cardiac arrest induced by local anaesthetic in a neonate.

In conclusion, massive ILE overdose can be fatal to a newborn, especially when the newborn is premature and hypotrophic or if the overdose occurs in the first postnatal hours in a patient whose vital functions are not fully stabilized. Healthcare professionals must be very vigilant when prescribing and administering ILE, especially when the ILE will be administered separately. The risk of progression to multiple organ failure, associated with extensive fat sludging in the microvasculature, should prompt ET at the first signs of clinical or biological worsening.

## Supplementary Information

**Additional file 1.**

## Data Availability

The dataset supporting the conclusions of this article is contained within the manuscript.

## Abbreviations

CPAP: nasal continuous positive airway pressure
ET: exchange transfusion
FiO_2_: fraction of inspired oxygen
ILE: intravenous lipid emulsion
NICU: neonatal intensive care unit
PH: pulmonary hypertension
PN: parenteral nutrition
